# Supplementary figures and images for: Linking extreme seasonality and gene expression in Arctic marine protists
Source: Sci Rep. 2023 Sep 5;13:14627. doi: 10.1038/s41598-023-41204-3 (PMC10480425; doi:10.1038/s41598-023-41204-3)

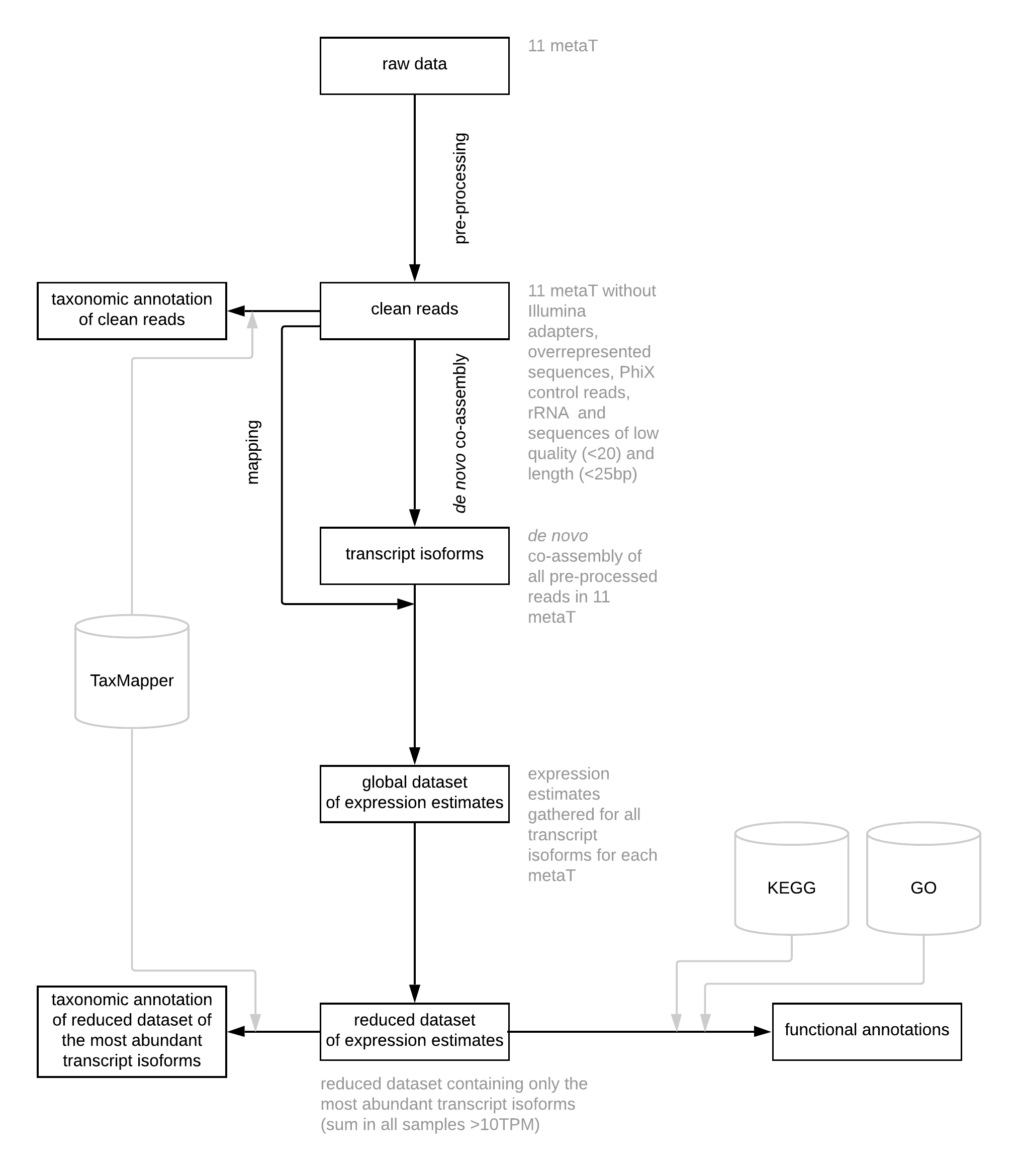

Supplement: Supplementary file 1 — Supplementary Information 1. [file 41598_2023_41204_MOESM1_ESM.png]

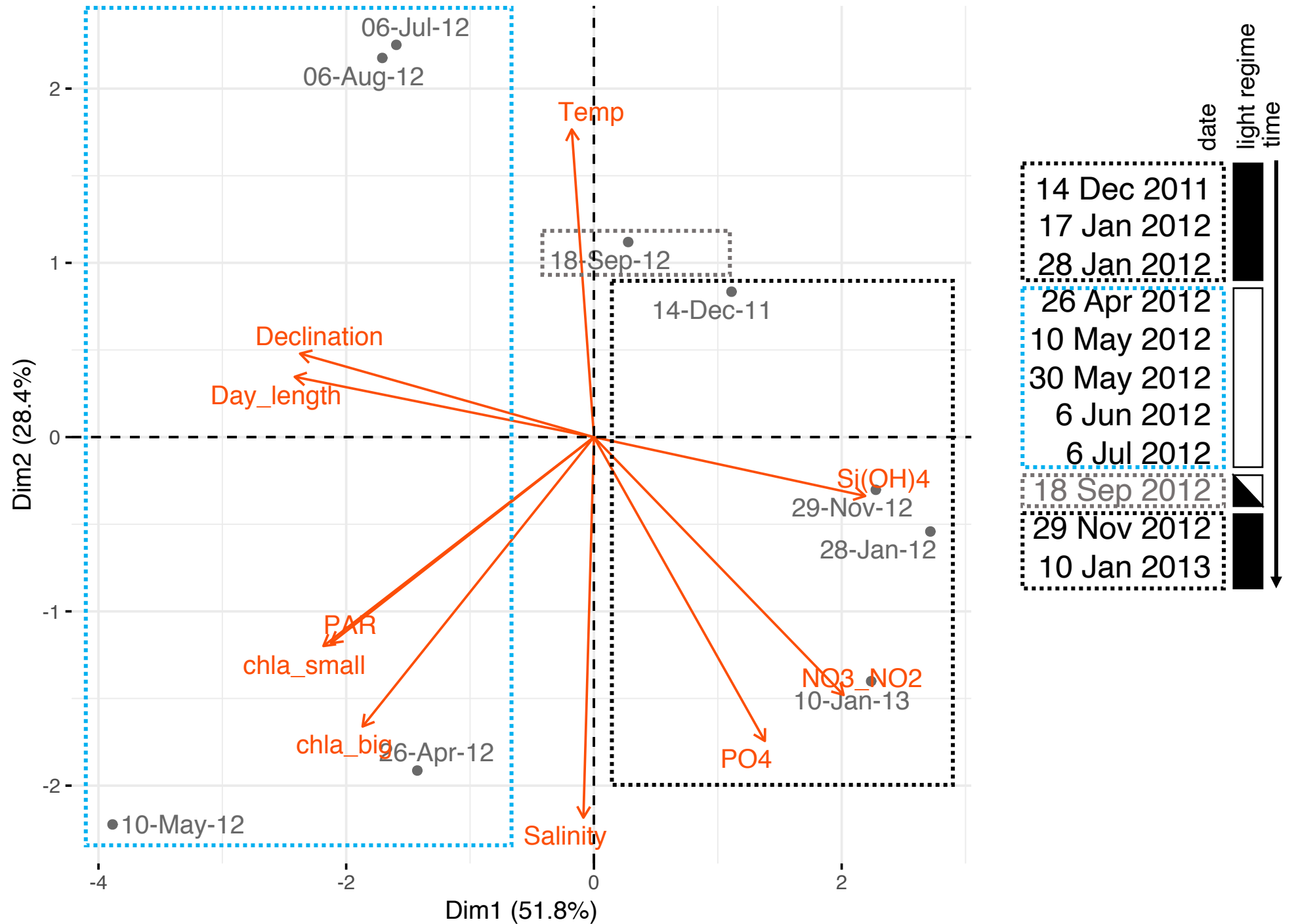

Supplement: Supplementary file 2 — Supplementary Information 2. [file 41598_2023_41204_MOESM2_ESM.pdf]

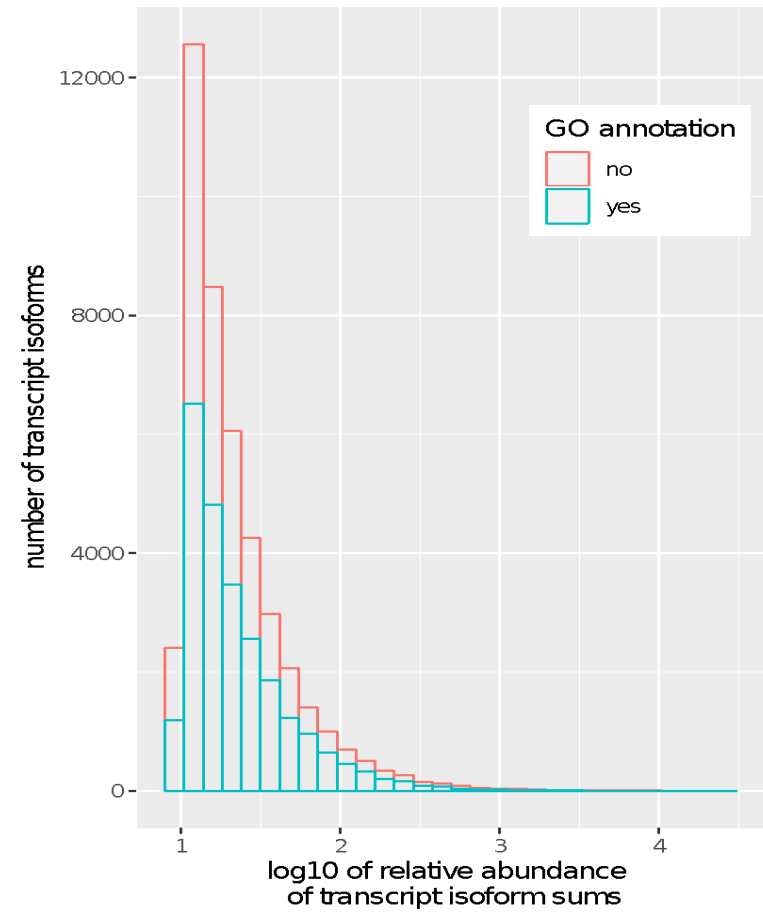

Supplement: Supplementary file 3 — Supplementary Information 3. [file 41598_2023_41204_MOESM3_ESM.pdf]
