# Supplementary material for: Linking extreme seasonality and gene expression in Arctic marine protists
Source: Sci Rep. 2023 Sep 5;13:14627. doi: 10.1038/s41598-023-41204-3 (PMC10480425; doi:10.1038/s41598-023-41204-3)
Supplement: Supplementary file 5 — Supplementary Information 5. [file 41598_2023_41204_MOESM5_ESM.docx]

1. grouping factor: polar night vs. polar day (without September)

average sd ratio ava avb cumulative sum

***TRINITY_DN3567435_c13_g1_i1 0.0091068 7.643e-03 1.1916 519.77 5236.75 0.01020***

*TRINITY_DN3531545_c4_g1_i13 0.0057171 4.039e-03 1.4154 645.86 3522.82 0.01661*

*TRINITY_DN3383058_c4_g1_i10 0.0049891 4.020e-03 1.2409 210.17 2793.45 0.02219*

*TRINITY_DN3531545_c4_g1_i11 0.0044515 2.692e-03 1.6536 572.16 2830.47 0.02718*

TRINITY_DN3528005_c14_g2_i1 0.0033457 1.859e-03 1.7995 51.95 1762.08 0.03093

*TRINITY_DN3567090_c10_g1_i2 0.0032716 2.241e-03 1.4599 411.10 2057.50 0.03459*

*TRINITY_DN3565854_c4_g3_i5 0.0032060 4.265e-03 0.7518 2.79 1660.42 0.03818*

TRINITY_DN3417822_c3_g2_i11 0.0031592 2.095e-03 1.5081 271.12 1859.83 0.04172

TRINITY_DN3313835_c6_g2_i3 0.0027240 2.927e-03 0.9306 45.65 1396.98 0.04477

*TRINITY_DN3417822_c3_g2_i2 0.0024838 2.791e-03 0.8898 397.70 1458.13 0.04756*

1. absence vs. presence of night

average sd ratio ava avb cumulative sum

***TRINITY_DN3567435_c13_g1_i1 0.0089522 0.0075762 1.1816 635.64 5236.74 0.01014***

*TRINITY_DN3531545_c4_g1_i13 0.0055438 0.0039526 1.4026 780.39 3522.82 0.01642*

*TRINITY_DN3383058_c4_g1_i10 0.0048617 0.0040257 1.2077 279.26 2793.45 0.02193*

*TRINITY_DN3531545_c4_g1_i11 0.0042184 0.0026293 1.6044 739.23 2830.47 0.02670*

TRINITY_DN3528005_c14_g2_i1 0.0033084 0.0018580 1.7807 74.10 1762.08 0.03045

*TRINITY_DN3565854_c4_g3_i5 0.0032128 0.0042582 0.7545 2.32 166.42 0.03409*

*TRINITY_DN3567090_c10_g1_i2 0.0031939 0.0022026 1.4501 469.78 2057.50 0.03771*

TRINITY_DN3417822_c3_g2_i11 0.0030590 0.0020509 1.4915 343.66 1859.83 0.04117

TRINITY_DN3313835_c6_g2_i3 0.0027182 0.0029221 0.9302 51.29 1396.98 0.04425

*TRINITY_DN3417822_c3_g2_i2 0.0024484 0.0027053 0.9050 478.65 1458.13 0.04702*

1. bloom vs. post-bloom

average sd ratio ava avb cumulative sum

***TRINITY_DN3567435_c13_g1_i1 0.0085442 4.672e-03 1.829 7807.2 1381.03 0.01159***

*TRINITY_DN3565854_c4_g3_i5 0.0056244 1.861e-03 3.022 11.80 4133.35 0.01922*

*TRINITY_DN3531545_c4_g1_i13 0.0048821 1.976e-03 2.471 4963.8 1361.30 0.02585*

*TRINITY_DN3383058_c4_g1_i10 0.0039800 2.688e-03 1.481 3964.9 1036.28 0.03125*

TRINITY_DN3561515_c5_g1_i4 0.0035883 2.845e-04 12.613 64.05 2689.11 0.03612

*TRINITY_DN3455354_c3_g3_i2 0.0031505 3.037e-04 10.376 24.96 2331.49 0.04039*

*TRINITY_DN3531545_c4_g1_i11 0.0031379 1.345e-03 2.334 3757.6 1439.78 0.04465*

TRINITY_DN3565854_c4_g3_i2 0.0028920 7.801e-04 3.707 7.66 2126.66 0.04857

*TRINITY_DN3567090_c10_g1_i2 0.0028720 1.036e-03 2.772 2911.7 776.19 0.05247*

TRINITY_DN3417822_c3_g2_i2 0.0028412 1.986e-03 1.4303 2269.4 241.16 0.05633
